# Elevated K^+^ channel activity opposes vasoconstrictor response to serotonin in cerebral arteries of the Fawn Hooded Hypertensive rat

**DOI:** 10.1152/physiolgenomics.00072.2016

**Published:** 2016-10-27

**Authors:** Mallikarjuna R. Pabbidi, Richard J. Roman

**Affiliations:** Department of Pharmacology and Toxicology, University of Mississippi Medical Center, Jackson, Mississippi

**Keywords:** serotonin (5-HT), myogenic response, K^+^ channels, calcium

## Abstract

Previous studies suggest that middle cerebral arteries (MCAs) of Fawn Hooded Hypertensive (FHH) rats exhibit impaired myogenic response and introgression of a small region of Brown Norway chromosome 1 containing 15 genes restored the response in FHH.1^BN^ congenic rat. The impaired myogenic response in FHH rats is associated with an increase in the activity of the large conductance potassium (BK) channel in vascular smooth muscle cells (VSMCs). The present study examined whether the increased BK channel function in FHH rat alters vasoconstrictor response to serotonin (5-HT). Basal myogenic tone and spontaneous myogenic response of the MCA was attenuated by about twofold and about fivefold, respectively in FHH compared with FHH.1^BN^ rats. 5-HT (0.1 μM)-mediated vasoconstriction was about twofold lower, and inhibition of the BK channel increased the vasoconstrictor response by about threefold in FHH compared with FHH.1^BN^ rats. 5-HT (3 μM) decreased BK channel and spontaneous transient outward currents in VSMCs isolated from FHH.1^BN^ but had no effect in FHH rats. 5-HT significantly depolarized the membrane potential in MCAs of FHH.1^BN^ than FHH rats. Blockade of the BK channel normalized 5-HT-induced depolarization in MCAs of FHH rats. The 5-HT-mediated increase in cytosolic calcium concentration was significantly reduced in plateau phase in the VSMCs of FHH relative to FHH.1^BN^ rats. These findings suggest that sequence variants in the genes located in the small region of FHH rat chromosome 1 impairs 5-HT-mediated vasoconstriction by decreasing its ability to inhibit BK channel activity, depolarize the membrane and blunt the rise in cytosolic calcium concentration.

cerebral vascular smooth muscle (VSMC) develops an intrinsic myogenic tone that maintains cerebral vessels in a partially constricted state (13). Vasoactive agents released from blood cells, platelets, the endothelium, astrocytes, and neurons modulate vascular tone to match cerebral blood flow (CBF) with local metabolic demand (10). Altered vascular reactivity to vasoactive agents, such as serotonin (5-HT), is associated with the pathogenesis of cardiovascular diseases, including hypertension, peripheral vascular disease, and migraines (41). 5-HT-mediated vascular constriction is enhanced in cerebral arteries isolated from rodent models of hypertension (5, 29).

5-HT acts on G_q_-coupled 5-HT_2A_ receptors and activates the phospholipase C (PLC) pathway (1, 27). PLC mediates phosphatidylinositol 4,5-bisphosphate breakdown to release inositol 1,4,5-trisphosphate (IP_3_) and diacylglycerol, which contribute to vascular constriction through IP_3_-mediated calcium release from intracellular stores and activation of protein kinase C (PKC) (38, 39). Large conductance K^+^ (BK) channels are initially activated by release of cytosolic calcium to generate spontaneous transient outward currents (STOCs) (2, 28). However, the rise in BK channel activity is opposed by progressive PKC-mediated phosphorylation, which eventually results in membrane depolarization and calcium entry via voltage-gated calcium channels (VGCC) (3, 45).

We have previously reported that the middle cerebral arteries (MCA) of the Fawn Hooded Hypertensive (FHH) rat exhibit impaired myogenic response to elevations in perfusion pressure and that introgression of a small region of BN (Brown Norway) chromosome 1 (FHH.1^BN^) containing 15 genes restores this response (31). The impaired myogenic response in the FHH rat is associated with an elevated BK channel activity compared with FHH.1^BN^ rats (6, 32). Functionally, the impaired myogenic response has been shown to impair autoregulation of CBF and greater infarct size following occlusion and reperfusion of the MCA in FHH rats (31). In addition, FHH rats have recently been reported to exhibit increased blood-brain barrier leakage, neurodegeneration, and loss of cognitive function following the development of hypertension (12). Sequence analysis revealed that there are >700 single nucleotide variants in 11 of the 15 genes located in the 2.4 Mbp region in FHH vs. FHH.1^BN^ rats. Only two genes (Mxi1 and Rbm20) were found to be differentially expressed in renal vessels isolated from these strains (6). Three of the genes in the region of interest (Add3, Rbm20, and Shoc-2) had sequence variants in the coding region that potentially could alter the function of the proteins. Of these genes, adducin 3 (Add3) appears to be a likely candidate for the altered K^+^ channel activity and the myogenic response, because Milan normotensive rats share the same variant in Add3 as FHH rats, and they also develop renal disease as they age (36, 40). More recently, we have reported that knockdown of the expression of Add3 impairs the myogenic response in the MCA of Sprague-Dawley rats and increases K^+^ channel activity (30).

Add3 dimerizes with Add1, and together they regulate actin-spectrin interactions (25). Potentially, a mutation in this gene might alter the cytoskeleton and the trafficking of ion channels. However, a prerequisite for testing whether a mutation in Add3 or any of the other genes in the 2.4 Mbp region are functionally significant is to determine under what experimental conditions K^+^ channel activity is elevated in VSMCs isolated from FHH vs. FHH.1^BN^ rats. Elevated K^+^ channel activity in the cerebral vasculature of FHH rats would be expected to alter vascular responsiveness to vasoconstrictors acting on G_q_-coupled receptors. Thus, the present study determined whether the cerebral vasculature of FHH rat exhibits impaired vasoconstrictor response to 5-HT that is associated with an inability to inhibit BK channel activity relative to FHH.1^BN^ rats, and if blockade of the BK channel can restore the vasoconstrictor response to 5-HT.

## MATERIALS AND METHODS

Experiments were performed on 72 nine- to 12-week-old male FHH and FHH.1^BN^ congenic rats that were obtained from inbred colonies maintained at the University Of Mississippi Medical Center (UMMC). The FHH chromosome 1 congenic strain was generated as previously described (22) by backcrossing FHH.1^BN^ consomic rats (26) to FHH rats to generate a F1 population (9). A F2 population with random recombination along chromosome 1, but homozygous for FHH alleles on all other chromosomes, was generated by intercrossing rats in the F1 population. The F2 rats were further backcrossed to FHH rats, and the pups were genotyped to narrow the region of interest and to generate overlapping FHH.1^BN^ congenic strains. The minimal FHH.1^BN^ congenic strain used in these studies has 2.4 Mbp region of BN chromosome 1 (RNO1) between markers D1Rat09 and D1Rat225 that contains 15 genes (6, 32). A genetic map comparing the 2.4 Mbp region of BN rat chromosome 1 introgressed into the FHH rat genetic background (FHH.1^BN^ congenic strain) was presented in previous studies (6, 19, 32). The rats were housed in the Animal Care Facility at UMMC, which is approved by the American Association for the Accreditation of Laboratory Animal Care. They had free access to food and water throughout the study. All protocols were approved by the Animal Care and Use Committee of UMMC.

### 

#### Measurement of basal tone, myogenic response, and vascular responsiveness to 5-HT in MCAs.

 These experiments were performed in MCAs isolated from FHH and FHH.1^BN^ rats that were euthanized with 4% isoflurane. The brain was removed and placed in a low-calcium solution containing (in mM): 145 NaCl, 4 KCl, 1 MgCl_2_, 10 HEPES, 0.05 CaCl_2_, and 10 glucose (pH 7.4). An unbranched segment of the MCA with an inner diameter of 100–200 μm was dissected. They were mounted on glass micropipettes, tied with 8-0 nylon in a myograph containing physiological salt solution (PSS) (in mM): 119 NaCl, 4.7 KCl, 1.17 MgSO_4_, 1.8 CaCl_2_, 18 NaHCO_3_, 5 HEPES, 1.18 NaH_2_PO_4_, and 10 glucose, pH 7.4. Calcium-free PSS was prepared by replacing CaCl_2_ with an equimolar concentration of MgCl_2_ and the addition of 2 mM EGTA. The inflow pipette was connected to a reservoir to allow for control of intraluminal pressure that was monitored with an in-line pressure transducer (Cobe, Lakewood, CO). The distal cannula was closed off so that there was no flow within the MCAs. A servo control system consisting of a miniature peristaltic pump and controller that permitted lumen pressure to be maintained at a constant pressure or increased at a defined rate (Living Systems Instrumentation). The cannulated MCAs were visualized using a charge-coupled device camera (DAGE MTI, CCD-72) mounted on an inverted microscope (Amscope, NI300 TB-FL). The inner diameter of the vessels was determined by a video dimension analyzer calibrated to a diameter of ± 2.0 μm (Living Systems Instrumentation). The bath solution was equilibrated with O_2_ (95%) and CO_2_ (5%) to provide adequate oxygenation and to maintain pH at 7.4. After mounting, the vessels were pressurized to 40 mmHg, and baseline diameter was measured at room temperature (22°C). Then, the bath temperature was increased to 37°C, and the development of basal tone was assessed by the change in diameter recorded over the next hour (equilibration period). After measuring basal developed tone, we increased pressure in a single step from 40 to 140 mmHg, and the myogenic response was determined as the change in diameter of the vessel over a 5 min period. Vasoconstrictor response to cumulative concentrations of 5-HT (10^−8^ to 10^−6^ M) was determined at a luminal pressure of 80 mmHg.

#### Isolation of cerebral VSMCs.

The rats were euthanized with 4% isoflurane. MCAs were microdissected and digested in a low-calcium dissociation solution containing (in mM): 145 NaCl, 4 KCl, 1 MgCl_2_, 10 HEPES, 0.05 CaCl_2_, and 10 glucose (pH 7.4). The vessels were cut into small pieces, spun down at 1,000 rpm for 1 min, and incubated in the dissociation solution containing papain (50 units or 2 mg/ml; Sigma, St. Louis, MO) and dithiothreitol (2 mg/ml) for 10–15 min at 37°C. The partially digested vessels were spun down at 1,000 rpm for 1 min, and the pellet was washed and resuspended in fresh dissociation solution containing albumin (1 mg/ml) collagenase (250 units/ml or 2 mg/ml) and incubated for 10–15 min at 37°C. The digested tissue was collected after centrifugation at 1,000 rpm, and the pellet was resuspended in dissociation solution. VSMCs were released into the media by gentle pipetting of the digested tissue. The supernatant was collected, and the cells were pelleted by centrifugation at 1,000 rpm for 1 min. The cells were resuspended in a low-Ca^2+^ dissociation solution and maintained at 4°C. Patch-clamp experiments were completed within 4 h after isolation of the cells.

#### Whole cell patch-clamp experiments.

K^+^ channel currents were recorded from VSMCs using a whole cell patch-clamp mode at room temperature (22–23°C). The bath solution contained (in mM): 130 NaCl, 5 KCl, 1.8 CaCl_2_, 1 MgCl_2_, 10 HEPES, and 10 glucose (pH 7.4). The pipettes were filled with a solution containing: 130 K-gluconate, 30 KCl, 10 NaCl, 1 MgCl_2_, and 10 HEPES (pH 7.2). The concentrations of EGTA and Ca^2+^ in the pipette solution were adjusted to obtain a cytosolic free Ca^2+^ concentration of 100 nM as determined using WinMAXC software written by C. Patton (Stanford University, Pacific Grove, CA; http://www.stanford.edu/∼cpatton/maxc.html). The pipettes were pulled from 1.5 mm (outer diameter) borosilicate glass capillaries using a two-stage micropipette puller (P-97; Sutter Instrument, San Rafael, CA) and heat-polished with a microforge. The pipettes had tip resistances of 2–8 MΩ. After the tip of a pipette was positioned on a cell, a 5–20 GΩ seal was formed, and the membrane ruptured by gentle suction with a glass syringe. An Axopatch 200B amplifier (Axon Instruments, Foster City, CA) was used to clamp the pipette potential and to record whole cell currents. Outward membrane K^+^ currents were elicited by a series of 20 mV voltage steps (from −60 to +120 mV) from a holding potential of −40 mV. The amplifier output signal was filtered at 2 kHz with an eight-pole Bessel filter. The currents were acquired using p-CLAMP software (version 10, Axon Instruments) at a rate of 10 kHz and stored on the hard disk of a computer for off-line analysis. Data analysis was performed using Clampfit software (version 10.0, Axon Instruments). Peak current amplitudes (pA) were determined from the average of 5–10 trials. Membrane capacitance, in picofarads (pF), was determined from the average of 30 currents measured in response to a 5 mV pulse. Peak currents were expressed as current density (pA/pF) to normalize for differences in the size of the VSMCs. Paxilline (Pax, 100 nM) and 4-aminopyridine (4-AP, 1 mM)-sensitive K^*+*^ channel currents were estimated from the total current after administration of 4-AP or Pax to the bath solution.

#### Perforated patch-clamp experiments.

Spontaneous transient outward currents (STOCs) were recorded by perforated patch-clamp technique in voltage clamp mode at a holding potential of +20 mV. Patch-clamp pipettes were made from borosilicate glass (Sutter Instruments, Novato, CA), coated with Sylgard to reduce capacitance and polished with a Micro Forge MF-830 fire polisher (Narishige Group, Tokyo, Japan). The final tip resistance averaged 4–6 MΩ. The extracellular solution contained (in mM): 134 NaCl, 6 KCl, 1 MgCl_2_, 2 CaCl_2_, 10 glucose, and 10 HEPES, and pH was adjusted to 7.4 with NaOH. The pipette solution contained (in mM): 110 potassium aspartate, 30 KCl, 10 NaCl, 1 MgCl_2_, 10 HEPES, and 0.05 EGTA. pH was adjusted to 7.2 with KOH and supplemented with freshly dissolved 150 μg/ml Nystatin. Recordings were started 5–10 min after the formation of the cell-attached patch configuration to allow adequate dialysis of the cells with the pipette solution. Currents were recorded using an Axopatch 200B amplifier, Digidata 1440A, and pCLAMP version 10.2 software (Molecular Devices, Union City, CA). Currents were sampled at 10 kHz and filtered at 2 KHz with a Bessel filter. STOCs were determined by counting the number of events recorded over a 5 min interval that was greater than a current threshold set at 2.5 times the single BK channel current amplitude for a particular holding potential. The frequency and mean amplitude of STOCs were determined off-line using Clampfit (version 10.0, Axon Instruments) and Origin Pro 9 software (OriginLab, Northampton, MA).

#### Protocol: microelectrode measurement of membrane potential in pressurized MCAs.

MCAs were mounted on glass micropipettes and pressurized to 80 mmHg at 37°C. The membrane potential (*V*_m_) was measured before and after administration of 5-HT (1 μM). The pipettes were pulled from 0.3 mm borosilicate glass capillaries using a two-stage micropipette puller (P-97; Sutter Instrument, San Rafael, CA). The pipettes had a tip resistances of 60–80 MΩ. The pipettes were backfilled with 3M KCl solution, and *V*_m_ was measured using electrometer (Intra 767 electrometer, WPI, Sarasota, FL). V_m_ signals were acquired using p-CLAMP software (version 10, Axon Instruments) at a rate of 10 kHz using Digidata 1440A (Molecular devices) and stored on the hard disk of a computer for off-line analysis. Data analysis was performed using Clampfit software (version 10.0, Axon Instruments).

#### Protocol: effects of 5-HT on cytosolic calcium.

VSMCs were freshly isolated from MCAs and allowed to attach to a 25 mm glass coverslip in a 1 ml perfusion chamber. The cells were bathed in physiological salt solution (PSS) containing (in mM): 130 NaCl, 5 KCl, 1.8 CaCl_2_, 1 MgCl_2_, 10 HEPES, and 10 glucose (pH 7.4). The cells were loaded with 5 μM fluo 4-AM (Molecular Probes) in PSS for 45 min in the dark at room temperature. cytosolic calcium ([Ca^2+^]_i_) was measured using a fluorescent inverted microscope (Nikon TS-100F) and Nikon NIS elements imaging system (Nikon). The cells were excited at wavelengths of 488 nm, and the images were recorded at emission wavelength of 510 nm by using ×40 objective. After baseline [Ca^2+^]_i_ was measured (F_o_), 5-HT was added to the bath, and [Ca^2+^]_i_ was recorded during a 4–5 min experimental period (F). The F/F_o_ values were obtained from multiple VSMCs and averaged to obtain a single value for statistical comparison.

#### Statistical analysis.

Data are expressed as mean values ± SE; *n* indicates the number of MCAs or VSMCs studied from different animals. The significance of differences in mean values was determined by Student's *t*-test for paired observations or one-way ANOVA followed by a Tukey's post hoc test for multiple comparisons. A *P* value <0.05 was considered to be significant.

## RESULTS

### 

#### Comparison of basal myogenic tone and the spontaneous myogenic response in MCAs isolated from FHH and FHH.1^BN^ rats.

**Fig. 1.**
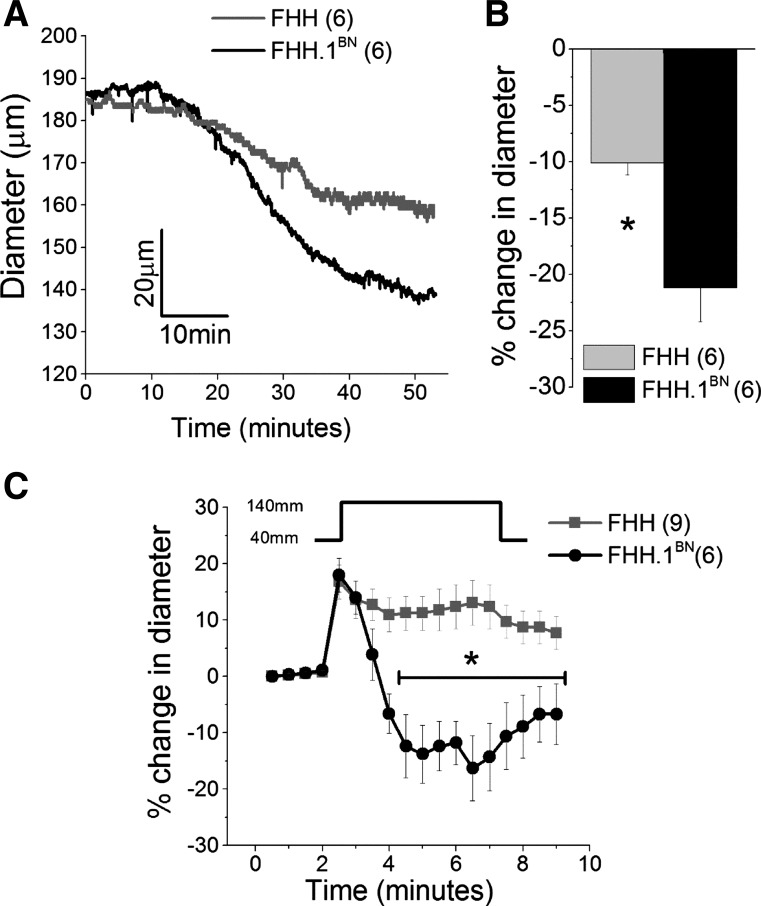
Comparison of basal tone and spontaneous myogenic response in middle cerebral arteries (MCAs) isolated from FHH and FHH.1^BN^ rats. MCAs were mounted on glass micropipettes in a myograph, and intraluminal pressure was maintained at 80 mmHg. *A* and *B*: traces and a comparison of the change in the diameter of the MCAs during equilibration period at 37°C. *C*: percent change in diameter of MCAs plotted over time in response to a step change in luminal pressure from 40 to 140 mmHg spontaneously. *Significant difference in the corresponding values in FHH and FHH.1^BN^ rats. Error bars ± SE. Numbers in parentheses indicate the number of vessels studied.

A comparison of the development of basal tone and the time course of the myogenic response in response to step change in pressure is presented in Fig. 1. MCAs isolated from FHH rats constricted by 8 ± 2% compared with 21 ± 3% in FHH.1^BN^ rats during equilibration period (22–37°C) (Fig. 1*B*, *n* = 6 vessels). A comparison of the time course of the myogenic response of MCAs isolated from FHH and FHH.1^BN^ rats in response to a single step change in transmural pressure from 40 to 140 mmHg is presented in Fig. 1*C* (*n* = 6–9 vessels). Immediately after we increased the pressure from 40 to 140 mmHg, the MCAs isolated from both strains distended to the same extent. During the next 4 min, MCAs of FHH.1^BN^ rats constricted to a level ∼15–20% below the baseline. In contrast, MCAs isolated from FHH rats did not respond and remained distended during this period (Fig. 1*C*). After pressure was returned to 40 mmHg, the MCA of FHH.1^BN^ rats dilated back to the baseline level, but the MCA of FHH rats became less distended (Fig. 1*C*).

#### Comparison of vascular response to 5-HT in MCAs of FHH and FHH.1^BN^ rats.

**Fig. 2.**
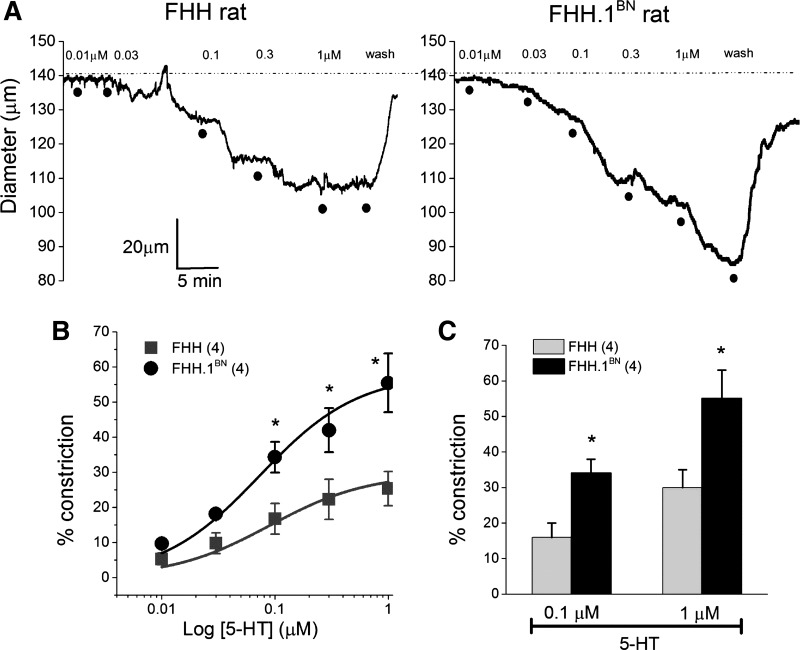
Comparison of serotonin (5-HT)-mediated vasoconstriction in MCAs isolated from FHH and FHH.1^BN^ rats. MCAs were mounted on glass micropipettes in a myograph, and the intraluminal pressure was maintained at 80 mmHg. After 1 h of equilibration at 37°C, 5-HT was applied to the vessel. *A*: representative diameter traces of 5-HT-mediated dose-dependent vasoconstriction in MCAs. *B*: percent constriction of MCAs plotted against log10 concentrations of 5-HT. The continuous lines are the best fit to the Hill equation. *C*: bar graph depicting the percent constriction in response to 0.1 and 1 μM 5-HT. *P < 0.05 between FHH and FHH.1^BN^ rats. Error bars ± SE. Numbers in parentheses indicate the number of vessels studied.

A comparison of the vasoconstrictor response to serotonin (5-HT) is presented in Fig. 2. 5-HT reduced the diameter of MCAs isolated from both FHH and FHH.1^BN^ rats in a concentration-dependent manner (Fig. 2*A*). However, the response to 5-HT was significantly greater in MCAs isolated from FHH.1^BN^ than FHH rats. E_max_ values averaged 42 ± 3% in FHH vs. 65 ± 9% FHH.1^BN^ rats (Fig. 2, *B* and *C*). EC_50_ values were not significantly different and averaged between 109 ± 7 and 107 ± 20 nM, respectively, in FHH and FHH.1^BN^ rats.

#### Effect of 5-HT on outward K^+^ channel currents in VSMCs isolated from FHH and FHH.1^BN^ rats.

**Fig. 3.**
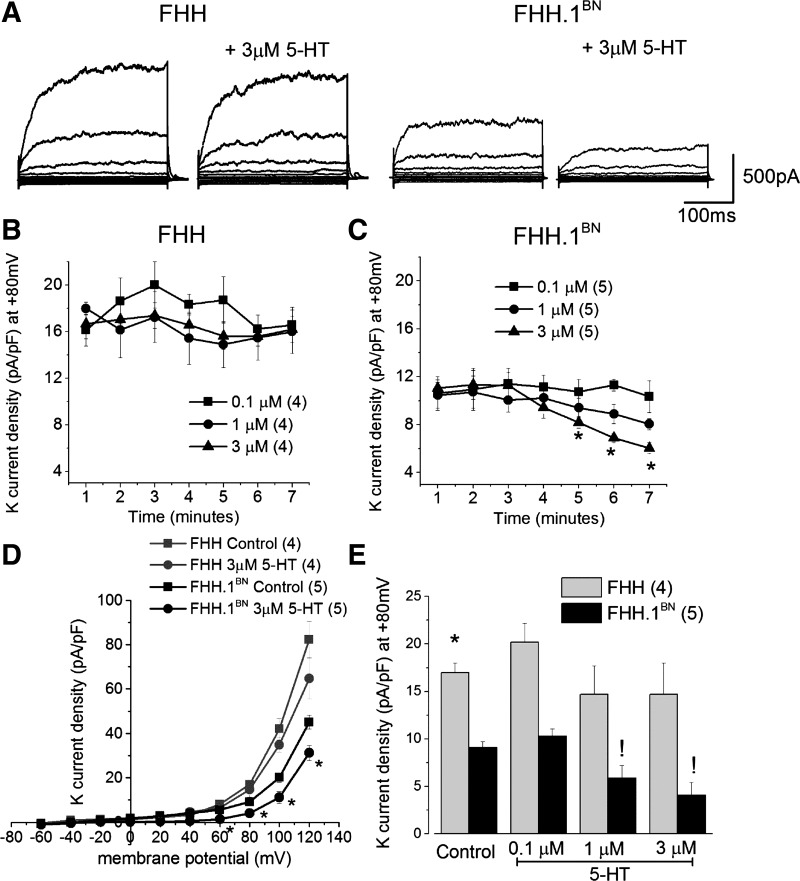
Comparison of 5-HT-mediated inhibition of outward potassium (K^+^) channel currents in vascular smooth muscle cells (VSMCs) isolated from MCAs of FHH and FHH.1^BN^ rats. Whole cell K^+^ channel currents were recorded with 0.1 μM free cytosolic calcium in the presence and absence of 5-HT. K^+^ channel currents were elicited by 20 ms pulses from −60 to +120 mV from a holding potential (*V*_h_) of −40 mV. *A*: whole cell total outward K^+^ channel currents before and after 3 μM 5-HT. *B* and *C*: changes in 5-HT-mediated K^+^ channel current density at +80 mV plotted over a period of 7 min. *D*: current-voltage relationship of the whole cell K^+^ channel current densities before and after 5-HT (4–6 cells). *E*: summary graph of current density at +80 mV before and after 0.3, 1, and 3 μM 5-HT (4–6 cells). *Significant difference in the corresponding values between FHH and FHH.1^BN^ rats; !significant difference in the corresponding values before and after 5-HT in FHH.1^BN^ rats. Error bars ± SE. Numbers in parentheses indicate the number of VSMCs studied.

Representative traces of K^+^ channel currents before and after administration of 3 μM 5-HT are shown in Fig. 3*A*. Baseline K^+^ channel currents were higher in VSMCs isolated from FHH compared with FHH.1^BN^ rats. Application of 5-HT (0.1–3 μM) for 7 min reduced K^+^ channel currents in FHH.1^BN^ rats but had no significant effect in cerebral VSMCs isolated from FHH rats (Fig. 3, *B* and *C*). The current-voltage curve was not altered by 3 μM 5-HT in FHH rats, but it was shifted to the right in FHH.1^BN^ rats (Fig. 3*D*). Total K^+^ channel current density measured at a holding potential of +80 mV in the presence of 1 and 3 μM 5-HT was significantly reduced in FHH.1^BN^ but not in FHH rats (Fig. 3*E*).

#### Effect of 5-HT on BK channel currents in VSMCs isolated from FHH and FHH.1^BN^ rats.

**Fig. 4.**
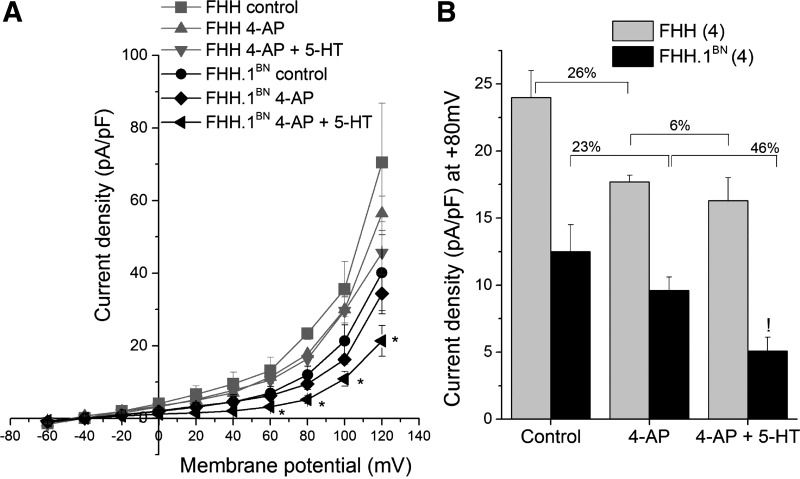
Comparison of 5-HT-mediated inhibition of large conductance potassium channel (BK) currents in VSMCs isolated from MCA of FHH and FHH.1^BN^ rats. Current-voltage curves of whole-cell BK currents in the presence and absence (control) of 4-aminopyridine (4-AP) combined with and without 3 μM 5-HT. *A*: K^+^ channel current densities plotted over membrane potential (4 cells). *B*: summary graph of current density at +80 mV (4 cells). *Significant difference in the corresponding values between FHH and FHH.1^BN^ rats; !significant difference in the corresponding values before and after 5-HT in FHH.1^BN^ rats. Error bars ± SE. Numbers in parentheses indicate the number of VSMCs studied.

To evaluate the relative contribution of the BK channel to the changes in total K^+^ currents VSMCs were treated with 1 mM 4-AP to block voltage-gated K^+^ channel (K_v_) currents, and the resulting BK channel currents were studied. The addition of 4-AP inhibited total K^+^ channel current similarly in VSMCs isolated from FHH and FHH.1^BN^ rats (26 vs. 23% inhibition) (Fig. 4*B*). 5-HT in the presence of 4-AP decreased the residual BK channel current significantly in FHH.1^BN^ rats but not in FHH rats (46 vs. 6%) (Fig. 4, *A* and *B*).

#### Comparison of the effects of 5-HT on STOCs in VSMCs isolated from FHH and FHH.1^BN^ rats.

**Fig. 5.**
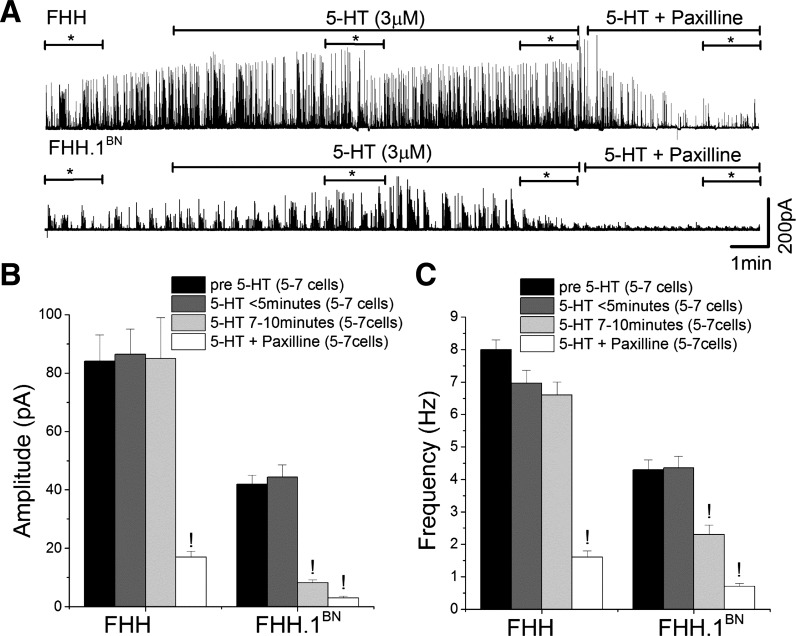
Comparison of 5-HT-mediated inhibition of spontaneous transient outward currents (STOCs) in VSMCs isolated from MCA of FHH and FHH.1^BN^ rats. STOCs were recorded at a *V*_h_ of +20 mV using perforated patch clamp method over a period of 7–10 min. *A*: traces of STOCs in the presence of 5-HT alone (3 μM) or combined with paxilline (100 nM). *B* and *C*: bar graph of STOC amplitude and frequency (*n* = 5–7 cells) measured before (pre-5-HT) and after (<5 min and between 7 and 10 min) application of 5-HT alone and combined with paxilline. *Region from where the amplitude and frequency of STOCs were measured. !Significant difference in the corresponding values before 5-HT application in FHH and FHH.1^BN^ rats. Error bars ± SE. Numbers in parentheses indicate the number of VSMCs studied.

VSMCs isolated from FHH rats exhibited a higher frequency and amplitude of STOCs compared with FHH.1^BN^ rats at a *V*_m_ of +20 mV (*n* = 7 and 5 cells, respectively; Fig. 5, *B* and *C*). The amplitude of STOCs decreased by ∼5-fold after 7–10 min of application of 5-HT to VSMCs isolated from FHH.1^BN^ rats. In contrast, 5-HT did not reduce the amplitude of STOCs in VSMCs isolated from FHH rats [(pA), FHH: 84 ± 9 to 84 ± 14; FHH.1^BN^: 43 ± 3 to 8 ± 1]. The frequency of STOCs fell by ∼50% in FHH.1^BN^ rats 7–10 min after administration of 5-HT, but it was not significantly altered in VSMCs isolated from FHH rats [(Hz), FHH: 8 ± 0.3 to 6.6 ± 0.4; FHH.1^BN^: 4.3 ± 3 to 2.3 ± 0.3, *P* < 0.05] (Fig. 5*C*). The amplitude and frequency of STOCs were markedly reduced in VSMCs obtained from both FHH and FHH.1^BN^ rats after administration of a BK channel inhibitor, Pax (Fig. 5).

#### Effect of 5-HT on V_m_ in pressurized MCAs isolated from FHH and FHH.1^BN^ rats.

**Fig. 6.**
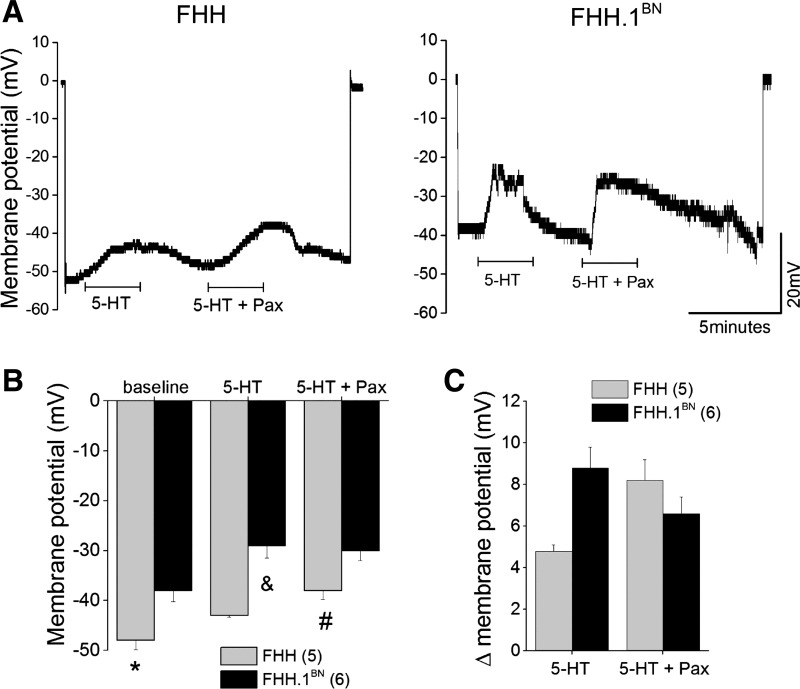
Comparison of 5-HT-mediated change in membrane potential in pressurized MCAs isolated from FHH and FHH.1^BN^ rats. Membrane potential was measured in pressurized (80 mmHg) MCAs using microelectrode impalement technique. *A* and *B*: traces of the original membrane potential recordings and summary bar graph of the membrane potential before and after application of either 5-HT (1 μM) alone or combined with paxilline (100 nM). *C*: delta change in the membrane potential before and after application of 5-HT alone or combined with paxilline. Values are means ± SE. *Significant difference in the corresponding values in FHH and FHH.1^BN^ rats; &significant difference in the corresponding values from the 5-HT-mediated depolarization in FHH.1^BN^ rats; #significant difference in the corresponding values from the 5-HT + paxilline-mediated depolarization in FHH rats. Numbers in parentheses indicate the number of vessels studied.

The results of these experiments are presented in Fig. 6. Baseline *V*_m_ was significantly more negative in FHH compared with FHH.1^BN^ rats (FHH: −48 + 1.8 mV; FHH.1^BN^: −38 ± 2.2 mV, *n* = 5–6 vessels; *P* < 0.05) (Fig. 6*B*). Application of 5-HT (1 μM) depolarized the *V*_m_ in both strains, but the magnitude of effect was significantly greater in FHH.1^BN^ than in FHH rats (delta *V*_m_ is 4.8 ± 0.3 in FHH rats vs. 8.8 ± 1 mV in FHH.1^BN^ rats) (Fig. 6*C*). Administration of the BK channel inhibitor Pax (100 nM) enhanced the membrane depolarization response to 5-HT in FHH to the same extent as seen in FHH.1^BN^ rats treated with 5-HT alone (Fig. 6C). In contrast, Pax had little effect on 5-HT-mediated depolarization in the MCA of FHH. 1^BN^ rats (Fig. 6*C*).

#### Effect of 5-HT on [Ca^2+^]_i_ in VSMCs isolated from FHH and FHH.1^BN^ rats.

**Fig. 7.**
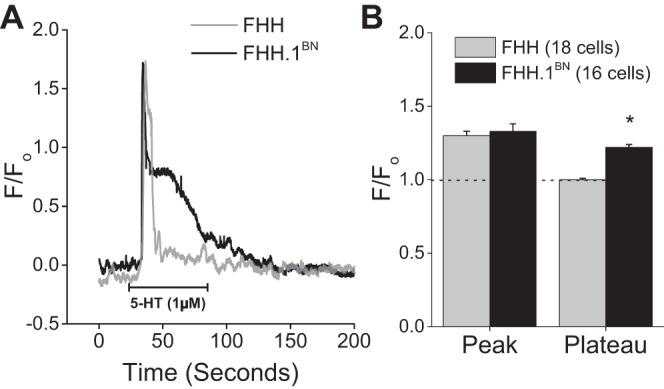
Comparison of 5-HT-mediated change in cytosolic calcium ([Ca^2+^]_i_) in VSMCs isolated from MCA of FHH and FHH.1^BN^ rats. VSMCs were isolated from MCAs and plated on 25 mm glass coverslip in a 1 ml perfusion chamber. We loaded 5 μM fluo 4-AM into the cells and measured [Ca^2+^]_i_. *A*: traces. *B*: summary graph of change in fluorescence during peak and plateau phases in response to 1 μM 5-HT. *Significant difference in the corresponding values in FHH and FHH.1^BN^ rats. Error bars ± SE. Numbers in parentheses indicate the number of VSMCs studied.

5-HT produced a biphasic increase in [Ca^2+^]_i_ in VSMCs isolated from both strains (Fig. 7*A*). The peak response to 5-HT was not significantly different in VSMCs isolated from FHH and FHH.1^BN^ rats. However, the plateau phase of the calcium response was significantly greater in FHH.1^BN^ than in the FHH rats (Fig. 7*B*).

#### Effect of BK channel blocker on contractile response to 5-HT.

**Fig. 8.**
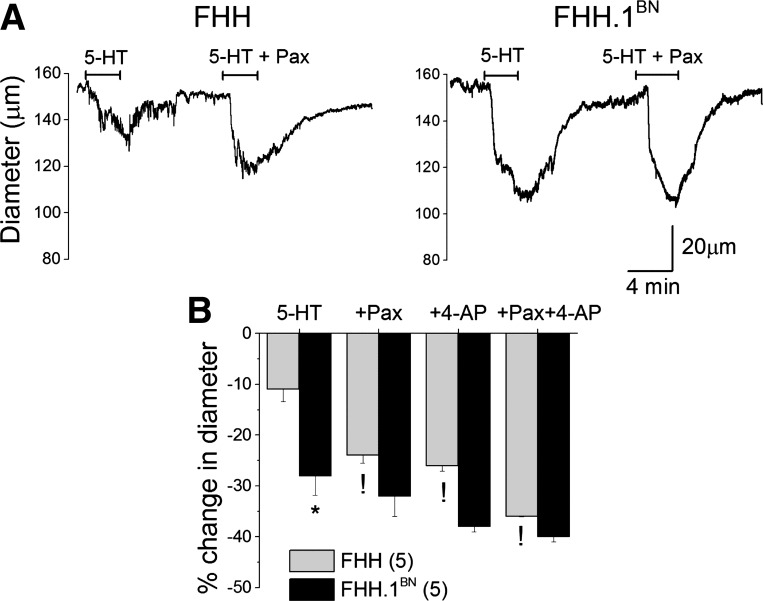
Comparison of 5-HT-mediated vasoconstriction in the presence of K^+^ channel inhibitors in MCAs isolated from FHH and FHH.1^BN^ rats. MCAs were mounted on glass micropipettes in a myograph, and intraluminal pressure was maintained at 80 mmHg. After 1 h of equilibration at 37°C, K^+^ channel blockers [4-AP, K_v_ channel blocker; Paxilline (Pax), BK channel blocker] combined with 100 nM 5-HT were applied to the chamber. *A*: diameter traces of 5-HT-mediated vasoconstriction in the presence or absence of 100 nM Pax. *B*: bar graph depicting the percent change in diameter (vasoconstriction) in the presence of 5-HT alone and in combination with BK channel blocker (+Pax, 100 nM) and/or K_v_ channel blocker (4-AP, 1 mM). *P < 0.05 between FHH and FHH.1^BN^ rats; !significant difference in the corresponding values after K channel inhibition compared with 5-HT-mediated vasoconstriction alone in FHH rats. Error bars ± SE. Numbers in parentheses indicate the number of vessels studied.

Administration of Pax (100 nM) had no significant effect on the diameter of MCAs isolated from FHH.1^BN^ rats, but it constricted MCAs isolated from FHH rats by ∼5% (data not shown). The vasoconstrictor response to 0. μM 5-HT was greater in the MCA of FHH.1^BN^ than in FHH rats. Blockade of BK channels with Pax enhanced the vasoconstrictor response to 5-HT in MCAs isolated from FHH rats, but it had no effect in vessels obtained from FHH.1^BN^ rats (Fig. 8). Administration of 4-AP to inhibit voltage-sensitive K_v_ channels, enhanced the vasoconstrictor response to 5-HT in FHH rats but had no effect in FHH.1^BN^ rats (Fig. 8*B*). The vasoconstrictor response to 5-HT was enhanced to a greater extent in the presence of both 4-AP and Pax in FHH rats, but it was not significantly altered in the MCA of FHH.1^BN^ rats (Fig. 8*B*).

## DISCUSSION

The present studies compared the vasoconstrictor response to 5-HT in MCAs isolated from FHH and FHH.1^BN^ rats. The results suggest that the basal vascular tone, the myogenic response to a step increase in pressure, and vasoconstrictor response to 5-HT are all significantly reduced in MCAs isolated from FHH rats compared with FHH.1^BN^ rats. The impaired constrictor response to 5-HT was associated with an increase in K^+^ channel activity in VSMCs isolated from FHH relative to FHH.1^BN^ rats. 5-HT induced a similar rapid rise in [Ca^2+^]_i_ in VSMCs isolated from both FHH and FHH.1^BN^ rats. However, the sustained increase in [Ca^2+^]_i_ in the plateau phase was significantly greater in FHH.1^BN^ than FHH rats. 5-HT depolarized the *V*_m_ in MCAs isolated from FHH.1^BN^ rats but had no significant effect in vessels isolated from FHH rats. 5-HT significantly depolarized *V*_m_ in vessels isolated from FHH rats after blockade of the BK channel with Pax but had less effect in the MCA of FHH.1^BN^ rats. In addition, blockade of the BK channel or K_v_ channel either alone or together significantly enhanced 5-HT-mediated vasoconstriction in MCAs isolated from FHH rats but had no effect on the response in vessels obtained from FHH.1^BN^ rats. These findings are consistent with the view that elevated K^+^ channel activity is responsible for the hyperpolarized *V*_m_ that blunts the [Ca^2+^]_i_ entry during plateau phase and attenuates the vasoconstrictor response to 5-HT in FHH rats.

### 

#### Diminished 5-HT-mediated vasoconstriction in MCAs of FHH rats is associated with an inability to inhibit K^+^ channel activity and less basal vascular tone.

Previous studies have indicated that elevations in the transmural pressure triggers a myogenic response in resistance vessels and reduces vascular diameter below the baseline (16, 17). Our previous studies suggest that pressure-mediated myogenic response in MCAs of FHH.1^BN^ rats is intact, but it is markedly impaired in MCAs of FHH rats (32). The impaired myogenic response in the MCA of FHH rats is associated with an elevation in BK channel function and STOCs in VSMCs isolated from this vessel (32). It is well known that 5-HT-mediated vasoconstriction is associated with inhibition of K_v_ and K_ATP_ channel activity in various vascular beds (4, 21, 37). Therefore, it is logical to hypothesize that FHH rats may exhibit a diminished vasoconstrictor response to 5-HT due to an inability to inhibit K^+^ channel function. Indeed, the results of the present study indicate that the vasoconstrictor response to 5-HT is markedly reduced in MCAs isolated from FHH relative to FHH.1^BN^ rats. Evaluation of the dose response curves indicates that the maximal response to 5-HT was attenuated in FHH rats compared with FHH.1^BN^ rats, but there were no significant difference in the EC_50_ values. This suggests that the differences in vascular reactivity are not related to the changes in the binding to the 5-HT receptors but rather to an alteration in postreceptor coupling to the downstream signal transduction pathways. Our results also suggest that 5-HT-mediated regulation of the K^+^ channel is both dose and time dependent and can be separated into two phases. We observed an initial K^+^ channel activation phase (<5 min) followed by an inhibitory phase (>5 min) at low doses of 5-HT (0.1 μM). The activation phase is greater in FHH than in FHH.1^BN^ rats (Fig. 3*D*). The initial activation of K^+^ channel is thought to be mediated by an IP_3_-mediated increase in [Ca^2+^]_i_ that is released from intracellular stores (20) or direct activation by IP_3_R1 (44). On the other hand, activation of PKC and translocation of phosphorylated forms of PKC to the membrane inhibit K^+^ channel activity and later contribute to inhibition of K^+^ channel activity (3, 45). In this regard, we found that high doses of 5-HT significantly inhibited K^+^ channels in FHH.1^BN^ rats but not in FHH rats. We also observed an enhanced 5-HT-mediated contraction in FHH rats in the presence of either BK or K_v_ channel inhibitors, which is consistent with the observation that greater BK and/or K_v_ channel activity contributes to diminished 5-HT-mediated vasoconstrictor response in FHH rats (Fig. 8). There was a fourfold increase in 5-HT-mediated contraction in FHH rats in the presence of both BK and K_v_ channel blockers compared with the twofold change in the presence of either drug alone (Fig. 8). This suggests that 5-HT only causes a partial blockade of the BK and K_v_ channels in FHH rats. The lack of effect of K^+^ channel inhibitors to increase 5-HT-mediated contraction in FHH.1^BN^ rats is consistent with the view that 5-HT alone can inhibit K^+^ channel activity in this strain.

#### Diminished 5-HT-mediated K^+^ channel inhibition in VSMCs of FHH rats may result in hyperpolarized sarcolemma that attenuates calcium influx [Ca^2+^].

There is an increasing evidence that agonist-induced vasoconstriction occurs in response to Ca^2+^ influx following depolarization of *V*_m_ (8, 15). The inability of 5-HT to inhibit K^+^ channel activity in FHH rats may result in the hyperpolarized *V*_m_. Indeed, *V*_m_ in pressurized MCAs averaged −38 ± 2 mV in FHH.1^BN^ vs. −48 ± 2 mV in FHH rats. In the presence of the BK channel blocker Pax, 5-HT significantly depolarized *V*_m_ in FHH vessels, but it had less effect in FHH.1^BN^ rats (Fig. 6). Thus, the data suggest that higher BK channel function in MCA of FHH rats hyperpolarizes the membrane that opposes Ca^2+^ influx through voltage-gated calcium channels (VGCC) and diminishes the vasoconstrictor response to 5-HT. Consistent with this interpretation, we found that the peak increase in [Ca^2+^]_i_ was similar in VSMCs isolated from both FHH.1^BN^ and FHH rats, but the “plateau phase” was diminished in FHH rats (Fig. 7). The initial peak increase in [Ca^2+^]_i_ in VSMC following administration of 5-HT is thought to be mediated by the IP_3_-mediated release of Ca^2+^ from intracellular stores, which is not altered by Ca^2+^ channel antagonists such as Co^2+^, La^3+^, or nifedipine (42). This is followed by a plateau phase that results from the opening of VGCCs or store-operated Ca^2+^ channels buffered by reuptake of Ca^2+^ into sarcoplasmic reticulum and mitochondria (33). Thus, a decrease in [Ca^2+^]_i_ in VSMCs of FHH rats may be due to hyperpolarization of the membrane that diminishes Ca^2+^ influx via VGCCs.

#### Mechanisms involved in 5-HT-mediated disinhibition of K^+^ channels in FHH rats.

The mechanism underlying the diminished ability of 5-HT to inhibit K^+^ channel activity in the cerebral vasculature of FHH rats remains to be determined. It normally activates PLC and triggers PKC-mediated inhibition of BK channels that contributes to VSMC membrane depolarization (3, 8). Any disruption in this pathway can alter 5-HT-mediated vasoconstriction. Thus, diminished 5-HT-mediated K^+^ channel inhibition in FHH rats could be related to an abnormality in BK channel inhibition. Internalization and a decrease in K_v_ channel activity via caveolin have also been suggested to enhance 5-HT-mediated vasoconstriction (8). Likewise, ANG II-mediated vascular contraction is associated with activation of PKCε and caveolin-dependent internalization of K_ATP_ channels (35). Along these lines, it is possible that disruption of caveolin-dependent internalization of K^+^ channels might contribute to the diminished vasoconstrictor response to 5-HT in the cerebral vasculature of FHH rats.

FHH rats and the congenic FHH.1^BN^ rat differ genetically by 15 genes in a 2.4 Mbp region of chromosome 1 (RNO1). Thus, the phenotypic differences observed in these strains are likely due to differences in the expression or function of one or more of the genes in this region. Sequence analysis revealed that there are about 791 single nucleotide variants in 11 of 15 genes located in the 2.4 Mbp region of FHH rats (6). Only two genes (Mxi1 and Rbm20) were found to be differentially expressed in renal vessels isolated from FHH vs. FHH.1^BN^ rats (6). However, there is no information available about whether these genes influence vascular function. Twenty variants were identified in exons of six genes in the region (6). Of these, six variants in four genes in this region, Add3, Dusp5, Shoc-2, and Rbm20, alter the amino acid structure of their respective proteins (6, 11, 32). Of these genes, the variants in Rbm20 are benign (6), but those in Add3, Dusp5, and Shoc-2 possibly can alter the function of these proteins.

Adducin (ADD) is a heterodimeric cytoskeleton protein consisting of an α-subunit (Add1) bound to either a β-(Add2) or γ-subunit (Add3). It promotes actin-spectrin interactions and inhibits actin polymerization by end-capping (25). Adducin is a major PKC and Rho kinase substrate (24). Phosphorylation of adducin by PKC or Rho-kinase promotes a redistribution of adducin from the membrane and changes the actin and spectrin capping and the cytoskeleton (7, 14, 24), which could influence ion-channel activity and the myogenic response (18, 34). In this regard, we have recently reported that knockdown of the expression of Add3 increases K^+^ channel activity and inhibits the myogenic response in cerebral arteries (30). However, it remains to be determined whether the mutation in Add3 in FHH is functionally significant and contributes to the impaired myogenic response and autoregulation of CBF in FHH rats.

DUSP5 (dual-specificity protein phosphatase) is another interesting candidate gene. Dusp5 is an enzyme involved in the dephosphorylation of PKC and ERK_1/2_ (23). The expression of Dusp5 protein is similar in the cerebral vasculature of FHH and FHH.1^BN^ congenic rats. However, the levels of phosphorylated PKC and ERK_1/2_ are markedly reduced in FHH vs FHH.1^BN^ rats (11). This is consistent with the view that the activity of Dusp5 might be elevated in FHH rats. However, knockdown of the expression of DUSP5 gene increases myogenic response in cerebral arteries of Sprague-Dawley rats (11, 43). Knockout of the Dusp5 gene in FHH.1^BN^ rats increased the myogenic response in cerebral arteries and autoregulation of CBF in vivo (11). This was associated with increased levels of phosphorylated PKC-β11 and ERK_1/2_ (11, 43).

More recently Hye Khan et al. (19) studied a possible role of aminopeptidase P gene (*Xpnpep1*) in vascular function. Aminopeptidase P gene is also located in the 2.4 Mbp region of RNO1, and the expression of this protein was elevated in cerebral vessels of FHH rats relative to FHH.1^BN^ rats. Higher levels of aminopeptidase P has been suggested to decrease bradykinin-(endothelium)-mediated vasodilation in FHH rats compared with FHH.1^BN^ rats (19). However, whether the genes located in the 2.4 Mbp region of RNO1 play a role in attenuated 5-HT-induced constriction in MCAs of FHH rats relative to FHH.1^BN^ rats is not known and warrants further investigation.

### Perspectives

The present study indicates that MCA isolated from FHH rats exhibit less basal tone, impaired myogenic response, and an attenuated vasoconstrictor response to 5-HT, which are coupled to disinhibition of BK and K_v_ channel activity. The impaired myogenic response and autoregulation of CBF in FHH rats may lead to increased transmission of lumen pressure to cerebral capillaries especially following the development of hypertension. Elevated capillary pressure can lead to disruption of blood-brain barrier, vascular leakage, cerebral inflammation, and neurodegeneration (12). The importance of our results is that suppression of BK and/or K_v_ channel currents is a common mechanism that is shared by vasoconstrictors such as 5-HT that activate G_q_-coupled receptors. Any disruption in the pathways that mediate this function could alter the vascular reactivity and the contractile state of the blood vessel.

## GRANTS

This work was supported in part by grants from UMMC and the American Heart Association (13SDG14000006) awarded to M. R. Pabbidi and National Heart, Lung and Blood Institute Grants RO1 HL-105997 and R01 HL-092105 awarded to R. J. Roman.

## DISCLOSURES

No conflicts of interest, financial or otherwise, are declared by the author(s).

## AUTHOR CONTRIBUTIONS

M.R.P. conception and design of research; M.R.P. performed experiments; M.R.P. analyzed data; M.R.P. and R.J.R. interpreted results of experiments; M.R.P. prepared figures; M.R.P. drafted manuscript; M.R.P. and R.J.R. edited and revised manuscript; M.R.P. and R.J.R. approved final version of manuscript.

## References

[1] Bae YM, Kim A, Kim J, Park SW, Kim TK, Lee YR, Kim B, Cho SI Serotonin depolarizes the membrane potential in rat mesenteric artery myocytes by decreasing voltage-gated K^+^ currents. Biochem Biophys Res Commun 347: 468–476, 2006. 16828462 10.1016/j.bbrc.2006.06.116

[2] Benham CD, Bolton TB Spontaneous transient outward currents in single visceral and vascular smooth muscle cells of the rabbit. J Physiol 381: 385–406, 1986. 2442353 10.1113/jphysiol.1986.sp016333 PMC1182985

[3] Bonev AD, Jaggar JH, Rubart M, Nelson MT Activators of protein kinase C decrease Ca^2+^ spark frequency in smooth muscle cells from cerebral arteries. Am J Physiol Cell Physiol 273: C2090–C2095, 1997. 10.1152/ajpcell.1997.273.6.C2090 9435516

[4] Bonev AD, Nelson MT Vasoconstrictors inhibit ATP-sensitive K^+^ channels in arterial smooth muscle through protein kinase C. J Gen Physiol 108: 315–323, 1996. 8894979 10.1085/jgp.108.4.315 PMC2229325

[5] Budzyn K, Ravi RM, Miller AA, Sobey CG Mechanisms of augmented vasoconstriction induced by 5-hydroxytryptamine in aortic rings from spontaneously hypertensive rats. Br J Pharmacol 155: 210–216, 2008. 18552867 10.1038/bjp.2008.247 PMC2538692

[6] Burke M, Pabbidi M, Fan F, Ge Y, Liu R, Williams JM, Sarkis A, Lazar J, Jacob HJ, Roman RJ Genetic basis of the impaired renal myogenic response in FHH rats. Am J Physiol Renal Physiol 304: F565–F577, 2013. 23220727 10.1152/ajprenal.00404.2012 PMC3602705

[7] Chen CL, Hsieh YT, Chen HC Phosphorylation of adducin by protein kinase Cdelta promotes cell motility. J Cell Sci 120: 1157–1167, 2007. 17341583 10.1242/jcs.03408

[8] Cogolludo A, Moreno L, Lodi F, Frazziano G, Cobeno L, Tamargo J, Perez-Vizcaino F Serotonin inhibits voltage-gated K+ currents in pulmonary artery smooth muscle cells: role of 5-HT2A receptors, caveolin-1, and KV15 channel internalization. Circ Res 98: 931–938, 2006. 16527989 10.1161/01.RES.0000216858.04599.e1

[9] Cowley AW Jr, Roman RJ, Jacob HJ Application of chromosomal substitution techniques in gene-function discovery. J Physiol 554: 46–55, 2004. 14678490 10.1113/jphysiol.2003.052613 PMC1664739

[10] Davis MJ, Hill MA Signaling mechanisms underlying the vascular myogenic response. Physiol Rev 79: 387–423, 1999. 10221985 10.1152/physrev.1999.79.2.387

[11] Fan F, Geurts AM, Pabbidi MR, Smith SV, Harder DR, Jacob H, Roman RJ Zinc-finger nuclease knockout of dual-specificity protein phosphatase-5 enhances the myogenic response and autoregulation of cerebral blood flow in FHH.1BN rats. PLoS One 9: e112878, 2014. 25397684 10.1371/journal.pone.0112878 PMC4232417

[12] Fan F, Pabbidi MR, Lin R, Ge Y, Gomez-Sanchez EP, Rajkowska G, Moulana M, Gonzalez-Fernandez E, Sims J, Elliott MR, Paul IA, Auchus AP, Mosley TH, Harder DR, Roman RJ Impaired myogenic response of MCA elevates transmission of pressure to penetrating arterioles and contributes to cerebral vascular disease in aging hypertensive FHH rats. FASEB J 30, Suppl 953.7, 2016.

[13] Faraci FM, Baumbach GL, Heistad DD Myogenic mechanisms in the cerebral circulation. J Hypertens Suppl 7: S61–S64, 1989. 2681598

[14] Fukata Y, Oshiro N, Kinoshita N, Kawano Y, Matsuoka Y, Bennett V, Matsuura Y, Kaibuchi K Phosphorylation of adducin by Rho-kinase plays a crucial role in cell motility. J Cell Biol 145: 347–361, 1999. 10209029 10.1083/jcb.145.2.347 PMC2133101

[15] Garland CJ The role of membrane depolarization in the contractile response of the rabbit basilar artery to 5-hydroxytryptamine. J Physiol 392: 333–348, 1987. 3446783 10.1113/jphysiol.1987.sp016783 PMC1192307

[16] Grande PO, Mellander S Characteristics of static and dynamic regulatory mechanisms in myogenic microvascular control. Acta Physiol Scand 102: 231–245, 1978. 626101 10.1111/j.1748-1716.1978.tb06067.x

[17] Greensmith JE, Duling BR Morphology of the constricted arteriolar wall: physiological implications. Am J Physiol Heart Circ Physiol 247: H687–H698, 1984. 10.1152/ajpheart.1984.247.5.H687 6496751

[18] Hill MA, Falcone JC, Meininger GA Evidence for protein kinase C involvement in arteriolar myogenic reactivity. Am J Physiol Heart Circ Physiol 259: H1586–H1594, 1990. 10.1152/ajpheart.1990.259.5.H1586 2240255

[19] Hye Khan MA, Sharma A, Rarick KR, Roman RJ, Harder DR, Imig JD Elevated aminopeptidase P attenuates cerebral arterial responses to bradykinin in fawn-hooded hypertensive rats. PLoS One 10: e0145335, 2015. 26683993 10.1371/journal.pone.0145335 PMC4686180

[20] Kim CJ, Weir BK, Macdonald RL, Zhang H Erythrocyte lysate releases Ca^2+^ from IP3-sensitive stores and activates Ca^2+^-dependent K^+^ channels in rat basilar smooth muscle cells. Neurol Res 20: 23–30, 1998. 9471099 10.1080/01616412.1998.11740480

[21] Kleppisch T, Nelson MT ATP-sensitive K^+^ currents in cerebral arterial smooth muscle: pharmacological and hormonal modulation. Am J Physiol Heart Circ Physiol 269: H1634–H1640, 1995. 10.1152/ajpheart.1995.269.5.H1634 7503259

[22] Lopez B, Ryan RP, Moreno C, Sarkis A, Lazar J, Provoost AP, Jacob HJ, Roman RJ Identification of a QTL on chromosome 1 for impaired autoregulation of RBF in fawn-hooded hypertensive rats. Am J Physiol Renal Physiol 290: F1213–F1221, 2006. 16303858 10.1152/ajprenal.00335.2005

[23] Mandl M, Slack DN, Keyse SM Specific inactivation and nuclear anchoring of extracellular signal-regulated kinase 2 by the inducible dual-specificity protein phosphatase DUSP5. Mol Cell Biol 25: 1830–1845, 2005. 15713638 10.1128/MCB.25.5.1830-1845.2005 PMC549372

[24] Matsuoka Y, Li X, Bennett V Adducin is an in vivo substrate for protein kinase C: phosphorylation in the MARCKS-related domain inhibits activity in promoting spectrin-actin complexes and occurs in many cells, including dendritic spines of neurons. J Cell Biol 142: 485–497, 1998. 9679146 10.1083/jcb.142.2.485 PMC2133059

[25] Matsuoka Y, Li X, Bennett V Adducin: structure, function and regulation. Cell Mol Life Sci 57: 884–895, 2000. 10950304 10.1007/PL00000731 PMC11146971

[26] Mattson DL, Kunert MP, Roman RJ, Jacob HJ, Cowley AW Jr Substitution of chromosome 1 ameliorates L-NAME hypertension and renal disease in the fawn-hooded hypertensive rat. Am J Physiol Renal Physiol 288: F1015–F1022, 2005. 15644486 10.1152/ajprenal.00374.2004

[27] Nagatomo T, Rashid M, Abul Muntasir H, Komiyama T Functions of 5-HT2A receptor and its antagonists in the cardiovascular system. Pharmacol Therapeut 104: 59–81, 2004. 10.1016/j.pharmthera.2004.08.005 15500909

[28] Nelson MT, Quayle JM Physiological roles and properties of potassium channels in arterial smooth muscle. Am J Physiol Cell Physiol 268: C799–C822, 1995. 10.1152/ajpcell.1995.268.4.C799 7733230

[29] Nishimura Y Characterization of 5-hydroxytryptamine receptors mediating contractions in basilar arteries from stroke-prone spontaneously hypertensive rats. Br J Pharmacol 117: 1325–1333, 1996. 8882632 10.1111/j.1476-5381.1996.tb16732.x PMC1909781

[30] Pabbidi MR, Ge Y, Harder DR, Roman RJ Role of gamma-adducin gene in cerebral vascular myogenic response. FASEB Journal Supplement 945.947, 2016.

[31] Pabbidi MR, Juncos J, Juncos L, Renic M, Tullos HJ, Lazar J, Jacob HJ, Harder DR, Roman RJ Identification of a region of rat chromosome 1 that impairs the myogenic response and autoregulation of cerebral blood flow in fawn-hooded hypertensive rats. Am J Physiol Heart Circ Physiol 304: H311–H317, 2013. 23144316 10.1152/ajpheart.00622.2012 PMC3543673

[32] Pabbidi MR, Mazur O, Fan F, Farley JM, Gebremedhin D, Harder DR, Roman RJ Enhanced large conductance K^+^ channel activity contributes to the impaired myogenic response in the cerebral vasculature of Fawn Hooded Hypertensive rats. Am J Physiol Heart Circ Physiol 306: H989–H1000, 2014. 24464756 10.1152/ajpheart.00636.2013 PMC3962634

[33] Putney JW Jr Capacitative calcium entry revisited. Cell calcium 11: 611–624, 1990. 1965707 10.1016/0143-4160(90)90016-n

[34] Randriamboavonjy V, Busse R, Fleming I 20-HETE-induced contraction of small coronary arteries depends on the activation of Rho-kinase. Hypertension 41: 801–806, 2003. 12623999 10.1161/01.HYP.0000047240.33861.6B

[35] Sampson LJ, Davies LM, Barrett-Jolley R, Standen NB, Dart C Angiotensin II-activated protein kinase C targets caveolae to inhibit aortic ATP-sensitive potassium channels. Cardiovasc Res 76: 61–70, 2007. 17582389 10.1016/j.cardiores.2007.05.020

[36] Sonoyama K, Greenstein AS, Micheletti R, Ferrari P, Schiavone A, Aghamohammadzadeh R, Withers SB, Tripodi G, Ferrandi M, Heagerty AM Mutation in the beta-adducin subunit causes tissue-specific damage to myogenic tone. J Hypertens 29: 466–474, 2011. 21150638 10.1097/HJH.0b013e328341e1a1

[37] Sung DJ, Noh HJ, Kim JG, Park SW, Kim B, Cho H, Bae YM Serotonin contracts the rat mesenteric artery by inhibiting 4-aminopyridine-sensitive Kv channels via the 5-HT2A receptor and Src tyrosine kinase. Exp Mol Med 45: e67, 2013. 24336234 10.1038/emm.2013.116 PMC3880459

[38] Tamir H, Hsiung SC, Yu PY, Liu KP, Adlersberg M, Nunez EA, Gershon MD Serotonergic signalling between thyroid cells: protein kinase C and 5-HT2 receptors in the secretion and action of serotonin. Synapse (New York, NY) 12: 155–168, 1992. 10.1002/syn.890120209 1336223

[39] Tasaki K, Hori M, Ozaki H, Karaki H, Wakabayashi I Difference in signal transduction mechanisms involved in 5-hydroxytryptamine- and U46619-induced vasoconstrictions. J Smooth Muscle Res 39: 107–117, 2003. 14695024 10.1540/jsmr.39.107

[40] Tripodi G, Szpirer C, Reina C, Szpirer J, Bianchi G Polymorphism of gamma-adducin gene in genetic hypertension and mapping of the gene to rat chromosome 1q55. Biochem Biophys Res Commun 237: 685–689, 1997. 9299427 10.1006/bbrc.1997.7173

[41] Villalon CM, Centurion D Cardiovascular responses produced by 5-hydroxytriptamine: a pharmacological update on the receptors/mechanisms involved and therapeutic implications. Naunyn-Schmiedebergs Arch Pharmacol 376: 45–63, 2007. 17703282 10.1007/s00210-007-0179-1

[42] Wang Y, Baimbridge KG, Mathers DA Effect of serotonin on intracellular free calcium of rat cerebrovascular smooth muscle cells in culture. Can J Physiol Pharmacol 69: 393–399, 1991. 2059904 10.1139/y91-060

[43] Wickramasekera NT, Gebremedhin D, Carver KA, Vakeel P, Ramchandran R, Schuett A, Harder DR Role of dual-specificity protein phosphatase-5 in modulating the myogenic response in rat cerebral arteries. J Appl Physiol 114: 252–261, 2013. 23172031 10.1152/japplphysiol.01026.2011 PMC3544499

[44] Zhao G, Neeb ZP, Leo MD, Pachuau J, Adebiyi A, Ouyang K, Chen J, Jaggar JH Type 1 IP3 receptors activate BKCa channels via local molecular coupling in arterial smooth muscle cells. J Gen Physiol 136: 283–291, 2010. 20713546 10.1085/jgp.201010453 PMC2931145

[45] Zhou XB, Wulfsen I, Utku E, Sausbier U, Sausbier M, Wieland T, Ruth P, Korth M Dual role of protein kinase C on BK channel regulation. Proc Natl Acad Sci USA 107: 8005–8010, 2010. 20385812 10.1073/pnas.0912029107 PMC2867903

